# Climatology and Mineral Waters of the United States

**Published:** 1886-07

**Authors:** 


CLIMATOLOGY AND MINERAL WATERS OF THE UNITED STATES. By A. N. Bell, A. M., M. D., Editor of *The Sanitarian*, member of American Medical Association, American Public Health Association, American Climatological Association, Medical Society of the State of New York, Kings County Medical Society, N. Y., Honorary Member of Connecticut Medical Society, Corresponding Member of the Epidermiological Society of London, formerly P. A. Surgeon U. S. Navy, etc. New York: William Wood & Company. 1885.

This book of 386 pages views the subjects which are treated of, more especially from a hygienic standpoint, and the reader will be somewhat disappointed in finding no attempt at elucidation of climatic influences upon the development of those phenomena which play so important a part in the etiology of cerebro-spinal meningitis, pernicious fevers, and that great scourge of our Southern seaports, yellow fever; yet there is much useful information embodied in it which commends it to all who are interested in sanitation.

